# Improving Timely Antibiotic Administration in Febrile Neutropenia: A Quality Improvement Initiative in a Tertiary Oncology Hospital

**DOI:** 10.7759/cureus.117668

**Published:** 2026-10-09

**Authors:** Gaurav Dalain, Vidit Kapoor, Prashant Mehta, Anshul Jain, Saphalta Baghmar, Pravas Mishra

**Affiliations:** 1 Medical Oncology, Amrita Institute of Medical Sciences and Research Centre, Faridabad, IND; 2 Hematology, Amrita Institute of Medical Sciences and Research Centre, Faridabad, IND

**Keywords:** febrile neutropenia, oncology emergency, plan-do-study-act, quality improvement, time to antibiotics

## Abstract

Background: Febrile neutropenia (FN) is an oncological emergency in which delays in the administration of empiric broad-spectrum antibiotics can increase the risk of sepsis and other serious complications.

Aim: This study aimed to reduce the median time from fever recognition to first-dose administration of empiric intravenous antibiotics to less than 60 minutes in adult oncology patients with FN presenting to the emergency department (ED) or inpatient department (IPD) of a tertiary oncology hospital.

Methods: This single-centre quality improvement (QI) study employed a prospective Plan-Do-Study-Act (PDSA) approach over six months in the ED and inpatient oncology ward of Amrita Institute of Medical Sciences, Faridabad, India. Oncology patients with fever and neutropenia were included, with 26 patients evaluated in each cycle. The intervention bundle comprised standardization of the FN definition and care pathway, triage prioritization of suspected FN, staff education, fever-alert chart flags, rapid clinician notification, streamlined antibiotic ordering and administration, vascular access escalation planning, and improved ward-level antibiotic availability. The primary process measure was median time from fever recognition to first-dose empiric intravenous antibiotic administration; the proportion of patients receiving antibiotics within 60 minutes was also assessed. Patients with missing or erroneous antibiotic-administration timestamps were excluded from these timeliness calculations but retained in the enrolled cohort used for all other measures.

Results: In the baseline cycle, the median time to antibiotic administration was 57.5 minutes, with 50% of patients receiving antibiotics within 60 minutes among those with valid time records; documentation errors were present in six of 26 cases. Following implementation of the intervention bundle, the median time decreased to 39.5 minutes in Cycle 2 and 39 minutes in Cycle 3, while the proportion treated within 60 minutes improved to 83.3% and 84%, respectively. Documentation quality also improved, with incomplete or erroneous time records decreasing from six cases at baseline to two in Cycle 2 and one in Cycle 3.

Conclusion: A structured, low-cost, multidisciplinary QI intervention was associated with faster and more reliable administration of empiric antibiotics in adult patients with FN. This project supports the use of PDSA-based workflow redesign, staff education, and rapid-alert systems to improve adherence to the 60-minute antibiotic target in routine oncology practice.

## Introduction

Febrile neutropenia (FN) is an oncologic emergency in patients receiving cytotoxic chemotherapy and is associated with a substantial risk of sepsis, intensive care admission, and death if treatment is delayed [1,2]. The clinical and economic burden of this complication is considerable: in an analysis of more than 41,000 adult cancer patients hospitalized with FN across 115 US medical centres, in-hospital mortality was 9.5%, with substantial associated length of stay and cost per admission [3]. In a prospective single-centre cohort study, each additional hour of delay in antibiotic administration was independently associated with an approximately 18% relative increase in 28-day mortality, providing direct empirical support for time-to-antibiotic as a modifiable quality-of-care target [4].

International guidelines, including those of the Infectious Diseases Society of America (IDSA) and the American Society of Clinical Oncology (ASCO), recommend prompt initiation of broad-spectrum empiric antibiotics, ideally within 60 minutes of fever recognition or emergency department (ED) triage, making time to antibiotics an important quality-of-care indicator in oncology practice [5,6]. However, real-world adherence to this benchmark remains suboptimal, particularly in acute care settings where rapid recognition and workflow activation are inconsistent [2,7].

Delays commonly occur when FN is not recognized early as a distinct chief complaint, when recent chemotherapy or immunocompromised status is not clearly identified at triage, or when triage priority is underestimated despite the risk of rapid clinical deterioration [2,7]. Additional barriers include delayed communication between nurses and physicians, non-standardized fever pathways, difficulty accessing indwelling central venous catheters, infusion-related issues, and competing demands in busy ED and inpatient department (IPD) settings, all of which can prolong time to antibiotic administration [2,8,9]. These challenges are further amplified in settings with frequent staff turnover, where sustaining adherence to time-sensitive protocols requires repeated education, standardization, and regular feedback [8,9].

Structured quality improvement (QI) initiatives have been reported to substantially reduce time-to-antibiotic delays in adult oncology emergency departments, although such programmes remain more widely described in pediatric than in adult oncology settings, and are sparsely reported from tertiary oncology centres in India and other low- and middle-income settings [10,11].

At our hospital, similar system-level gaps were identified across both the ED and IPD, prompting development of this QI initiative, structured using the Plan-Do-Study-Act (PDSA) model for improvement [12], to improve timely antibiotic administration in adult oncology patients with FN. The project focused on standardizing the definition of FN, recognizing FN as a distinct chief complaint at ED presentation, revising the ED triage level for suspected FN, and implementing a bundled workflow comprising staff education, real-time fever alerts, rapid notification of physicians and nurses, and improved antibiotic and vascular access processes. The primary aim was to reduce the median time from fever detection to first-dose broad-spectrum intravenous antibiotics to less than 60 minutes in both the emergency department and oncology ward, while generating locally relevant evidence for feasible, low-cost QI strategies in a tertiary oncology hospital.

## Materials and methods

Study design and setting

This was a prospective, single-centre QI study employing a PDSA framework with audit-based evaluation, conducted in the ED and inpatient oncology ward of Amrita Institute of Medical Sciences, Faridabad, a tertiary care hospital in North India. The study reflects routine institutional clinical practice and was designed to improve the timeliness of empiric antibiotic administration in oncology patients presenting with FN, without introducing any experimental diagnostic or therapeutic agent.

Study duration

The project was conducted over six months, structured as three sequential PDSA cycles of approximately two months each: a baseline audit (Cycle 1), a first intervention cycle following introduction of the protocol bundle (Cycle 2), and a consolidation cycle following reinforcement of training (Cycle 3).

Sampling technique and sample size

All consecutive adult oncology patients meeting the case definition of FN who presented to the ED or were already admitted to the inpatient oncology ward during each cycle period were enrolled using a consecutive (total population) sampling approach; no random selection was performed. As this was a process-improvement audit rather than a hypothesis-testing study, no formal a priori sample size or power calculation was undertaken. The sample of 26 patients per cycle was determined pragmatically, based on the anticipated number of FN presentations at the study site over each cycle period, to allow meaningful cycle-to-cycle comparison of process measures within the six-month project timeline. A total of 78 patients were enrolled across the three cycles.

Inclusion and exclusion criteria

Inclusion criteria were adult patients (age ≥18 years) with an underlying malignancy receiving active cytotoxic chemotherapy who met the pre-specified definition of FN (below) at presentation to the ED or during inpatient admission. Exclusion criteria were age <18 years; fever without neutropenia, or neutropenia without fever; patients who had already received empiric antibiotics at an outside facility prior to arrival, precluding accurate measurement of institutional time to antibiotics; and patients in whom a diagnosis of FN could not be confirmed on subsequent case-sheet review.

FN was defined per the IDSA/ASCO criteria as a single oral temperature ≥38.3°C (101°F) or a temperature ≥38.0°C (100.4°F) sustained over a one-hour period, in the presence of an absolute neutrophil count (ANC) <500 cells/mm³, or an ANC <1000 cells/mm³ with an anticipated decline to <500 cells/mm³ within the next 48 hours.

Intervention

A standardized FN management protocol based on IDSA/ASCO guidance was introduced, supported by staff training, fever-alert chart flags, prioritization of FN as a distinct chief complaint at emergency triage, streamlined notification and antibiotic-ordering workflows, and assured ward-level antibiotic availability. Full implementation details, including timing, responsible staff, sequencing, and adherence assessment for each component, are provided in the Appendix. 

Data collection and outcome measures

Data were collected daily using a structured checklist and reviewed through clinical audit. The primary process measure was median time from fever recognition to first-dose empiric broad-spectrum intravenous antibiotic administration, with a target of less than 60 minutes; cases were also categorized as on-time or delayed according to this institutional benchmark. Secondary measures included completeness of case-sheet documentation (time-stamp accuracy) and qualitative staff feedback regarding barriers to implementation, gathered during PDSA team debriefs. Of the 26 patients enrolled per cycle, the denominator for the primary timeliness outcome (median time to antibiotic and proportion treated within 60 minutes) was restricted to patients with a complete and internally consistent fever-recognition and antibiotic-administration timestamp pair. Patients with a missing, duplicate, or implausible timestamp (e.g., a recorded antibiotic time preceding the recorded fever-recognition time) could not have an interval calculated; these cases were excluded from the timeliness denominator using a complete-case approach but were retained in the total enrolled cohort and counted toward the documentation-error rate, a separate secondary measure. A documentation error was defined as a record with a missing, duplicate, or implausible fever recognition or antibiotic administration time stamp, such as an antibiotic time earlier than the fever time, so that the interval could not be calculated.

Definition of time points

The outcome interval was calculated, in minutes, as the time of first-dose antibiotic administration minus time zero (fever recognition). Time zero was defined as the earliest documented time at which fever meeting the study definition was objectively identified by healthcare staff, namely, the timestamp of the first measured oral temperature ≥38.3°C (or the first of the readings ≥38.0°C sustained over one hour) recorded in the ED triage record or the ward nursing chart. Patient- or caregiver-reported fever without a measured temperature was not used to define time zero. For patients presenting to the ED, time zero was the time of the qualifying temperature recorded at triage, not the registration or arrival time; for inpatients, it was the nursing-chart time of the first qualifying temperature. Where the one-hour sustained-fever criterion applied, time zero was the time of the first elevated reading/the time at which the one-hour criterion was met. Time zero was not delayed until the ANC result became available, so that patients with a pending neutrophil count were timed from the fever reading. The end point, time of antibiotic administration, was defined as the time at which the first dose of the empiric intravenous antibiotic was started, as recorded by the administering nurse in the medication administration record; the time of the antibiotic order and the time of pharmacy dispensing were not used. Time points were abstracted to the nearest minute from paper case sheets/electronic records. 

Confounding variables

As an observational, audit-based QI initiative without randomization or a concurrent control arm, this study was susceptible to several potential confounders that were not formally adjusted for but are acknowledged qualitatively: time of presentation (day versus night shift, weekday versus weekend), ED patient census and triage acuity mix at presentation, ease of vascular access (functioning indwelling catheter versus need for peripheral access), seniority and experience of on-duty clinical staff, site of presentation (ED versus inpatient ward), and concurrent hospital-wide patient load. Because sequential PDSA cycles were also accompanied by increasing staff familiarity with the audit process, some of the observed improvement may partly reflect heightened awareness of being observed (a Hawthorne-type effect) in addition to the intervention components themselves.

Statistical analysis

Data were analyzed using descriptive statistics. Continuous variables (time to antibiotic administration) were summarized as medians, given the expected right-skewed distribution of time-based clinical data; categorical variables (proportion of patients treated within 60 minutes, documentation error rate) were summarized as frequencies and percentages. Between-cycle differences are presented descriptively, consistent with standard reporting conventions for PDSA-based QI projects. Analyses were performed using Microsoft Excel for Microsoft 365 (Microsoft Corp., USA). 

Ethical considerations

This project was reviewed and approved by the Institutional Ethics Committee of Amrita Institute of Medical Sciences, Faridabad (approval no. ECR/1 873/lnst/HRl2A23). As a QI initiative auditing routinely collected, de-identified clinical process data rather than testing an experimental intervention, a waiver of individual informed consent was granted. Patient confidentiality was maintained throughout data collection and analysis in accordance with institutional data-protection policy.

## Results

Across the three cycles, 26 patients were enrolled per cycle (78 in total); the primary timeliness outcome was calculated among the subset with valid antibiotic-administration timestamps (20, 24, and 25 patients in Cycles 1, 2, and 3, respectively), as detailed in the Methods. In Cycle 1 (baseline), the median time from fever recognition to empiric antibiotic administration was 57.5 minutes, with only 10 (50%) of 20 patients receiving antibiotics within the 60-minute target and the remaining 10 (50%) patients experiencing delays beyond 60 minutes. Among those delayed, the median time to antibiotics was prolonged at approximately 279.5 minutes (~4.7 hours), and documentation quality was suboptimal, with six (23.1%) of 26 patients having incomplete or erroneous time records.

Following implementation of the standardized fever protocol, staff training, fever-alert system, and improved ward-level antibiotic stock, Cycle 2 showed marked improvement. The median time to antibiotic administration decreased to 39.5 minutes, and the proportion of patients treated within 60 minutes increased to 20 (83.3%) of 24 patients, with four (16.7%) patients delayed beyond 60 minutes. The median time among delayed patients fell to approximately 130 minutes, and documentation errors reduced to two (7.7%) of 26 patients (Figure 1), indicating more reliable data capture and process adherence.

Further reinforcement of protocols and staff training was undertaken after Cycle 2, and gains were sustained in Cycle 3, where the median time to antibiotics remained low at 39 minutes and 21 (84%) of 25 patients received antibiotics within 60 minutes, with only four (16%) patients being delayed. The median delay among the small group still exceeding 60 minutes was approximately 150 minutes, and documentation errors declined further to one (3.8%) of 26 patients (Figure 1).

**Figure 1:**
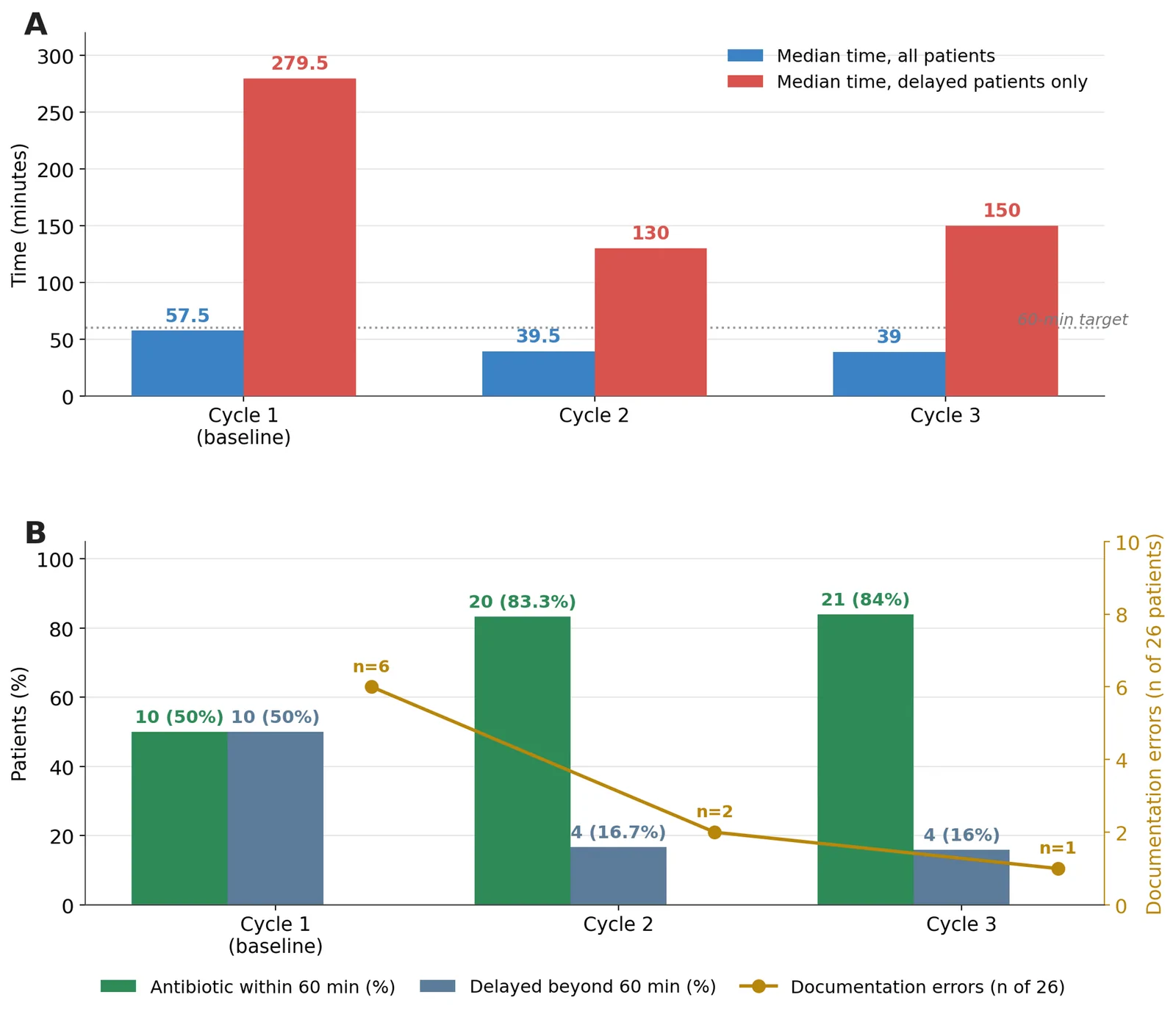
A: Median time to antibiotic administration across Plan-Do-Study-Act (PDSA) Cycles 1–3. B: Proportion of patients treated within 60 minutes, proportion delayed beyond 60 minutes, and documentation errors across PDSA Cycles 1–3.

The proportion treated within 60 minutes was 50% (95% CI, 27.2%-72.8%) in Cycle 1, 83.3% (95% CI, 62.6%-95.3%) in Cycle 2, and 84% (95% CI, 63.9%-95.5%) in Cycle 3. The absolute increase from Cycle 1 to Cycle 3 was 34.0 percentage points (95% CI, 6.6-56.2).

Table 1 summarizes the primary process measures across the three PDSA cycles. Figure 1 illustrates the corresponding trends.

**Table 1:** Primary process measures across the three Plan-Do-Study-Act (PDSA) cycles. Percentages for antibiotic administration within 60 minutes were calculated using patients with valid time entries as the denominator. Documentation errors are reported for all enrolled patients per cycle.

Cycle	Total patients	Valid time entries	Documentation errors, n	Median time to antibiotic (min)	Antibiotic ≤60 min, n (%)	Delay >60 min, n (%)	Median time among delayed, min
Cycle 1	26	20	6	57.5	10 (50%)	10 (50%)	279.5
Cycle 2	26	24	2	39.5	20 (83.3%)	4 (16.7%)	130
Cycle 3	26	25	1	39	21 (84%)	4 (16%)	150

## Discussion

These findings are consistent with current febrile neutropenia guidelines and prior QI literature emphasizing the importance of immediate empirical therapy and streamlined care pathways [5,6,8,9]. The IDSA guideline states that immediate empirical antibiotic therapy remains a core principle in neutropenic fever management, while the ASCO/IDSA update endorses administration of the initial empirical antibacterial dose within one hour of triage for eligible patients [5,6]. QI studies in pediatric oncology have similarly shown that protocolized workflows, staff education, and standardized escalation processes can shorten time to antibiotics in febrile neutropenia [8,9].

The magnitude of improvement achieved in this project is broadly comparable to other published adult-focused febrile neutropenia QI initiatives. Keng et al. reported a reduction in median time to antibiotics from 235 minutes in a historical cohort to 81 minutes following implementation of a structured emergency department febrile neutropenia pathway, with just under one-third of patients meeting a 60-minute benchmark [10]. Bruce similarly described a QI initiative targeting time-to-antibiotic administration for febrile neutropenia in a community hospital emergency department [11]. The median times achieved in the present project (57.5 minutes at baseline, improving to 39.5 and 39 minutes in Cycles 2 and 3) and the proportion of patients meeting the 60-minute benchmark (improving from 50% to 84%) compare favourably with these previously published adult series, suggesting that a bundled, low-resource intervention can achieve gains at least comparable to those reported from higher-resource settings.

The rationale for pursuing a 60-minute time-to-antibiotic target rests on evidence linking delay to adverse outcomes, although this evidence is not entirely consistent. In the large multicentre analysis described above, febrile neutropenia carried an in-hospital mortality of 9.5% [3], and a prospective single-centre cohort found that each additional hour of delay in antibiotic administration was independently associated with an approximately 18% relative increase in 28-day mortality [4]. However, not all studies confirm this association: in a retrospective cohort of 3,219 febrile neutropenia admissions, time to antibiotics was not significantly associated with 30-day mortality on multivariate analysis, although earlier administration was associated with a modestly shorter hospital stay [13]. This inconsistency across studies likely reflects heterogeneity in patient risk profiles, malignancy type, and case mix, and underscores that time-to-antibiotic is best interpreted as a process-quality indicator rather than a guaranteed determinant of mortality in every patient population. As the present study was designed to evaluate process measures only, it cannot speak directly to this outcomes literature; this is discussed further as a limitation below.

The improvement observed in this project appears to have resulted from addressing several system barriers simultaneously. A key early barrier was delayed recognition of immunocompromised status at ED presentation. To address this, patients and caregivers were educated to clearly communicate recent chemotherapy exposure at arrival, and triage staff were guided to prioritize these patients. A second barrier was limited situational awareness among physicians and nurses when an oncology patient with fever arrived. The bundled pathway therefore emphasized immediate notification of the treating team, rapid initiation of a standardized order set for cultures and empiric antibiotics, and prompt assessment of the indwelling catheter or alternative venous access.

Operational barriers related to vascular access also appeared important. Introduction of an explicit escalation algorithm permitting prompt peripheral intravenous access when the indwelling catheter could not be rapidly accessed likely reduced avoidable delay. In parallel, unit nurse champions were identified, and a senior oncologist conducted focused teaching sessions for emergency and inpatient staff to emphasize the time-sensitive nature of febrile neutropenia, the mortality risk associated with delayed treatment, and the need for a bundled cultures-plus-antibiotics approach.

The sustained gains observed in Cycle 3, following reinforcement during the second PDSA cycle, suggest that recurrent protocol-based training is essential for maintaining timely febrile neutropenia care in settings with frequent staff turnover. The temporal pattern of improvement, with the most marked gain after Cycle 1 and maintenance through Cycle 3, suggests that these measures improved both awareness and workflow reliability.

The decline in documentation error across cycles suggests that the checklist and protocol were increasingly integrated into routine practice and that staff engagement with the project improved over time. This indicates better recording of process times, which reduces the missing-data problem, but it does not by itself establish that the observed reduction in time to antibiotics represents genuine process improvement.

Several limitations should be acknowledged. This was a single-centre initiative with relatively small numbers per cycle, which limits statistical precision and external generalizability. The project focused on a process outcome rather than patient-centered clinical endpoints such as ICU admission, septic shock, mortality, or length of hospital stay; as summarized above, the literature relating time-to-antibiotic to such hard outcomes is itself mixed, and this project cannot resolve that debate. In addition, because the intervention was delivered as a bundle, the individual contribution of each component cannot be isolated. Residual delays in Cycles 2 and 3 also indicate that some workflow barriers persisted despite improvement. The primary timeliness outcome also relied on a complete-case denominator that excluded patients with missing or erroneous timestamps (6, 2, and 1 patients in Cycles 1-3, respectively); if these exclusions were not unrelated to actual antibiotic timing, the reported proportions treated within 60 minutes could be biased, and this assumption could not be formally tested given the small numbers involved. Patient and presentation characteristics (e.g., site and time of presentation, clinical acuity, vascular access status) were not compared across cycles, so changes in case mix cannot be excluded as a contributor to the improvement; the findings should be interpreted as an association with the bundled intervention rather than evidence of its effect.

Despite these limitations, the project has important practical implications. It demonstrates that meaningful improvement in time to antibiotic administration can be achieved in a resource-constrained oncology setting through low-cost, workflow-focused interventions grounded in guideline-based care [5,6]. The findings suggest that delay in febrile neutropenia is rarely attributable to a single failure point and is better addressed through a coordinated pathway incorporating early recognition, rapid triage, immediate clinician notification, predefined order sets, contingency planning for vascular access, nursing leadership, and reliable ward-level antibiotic availability. The sustained performance in Cycle 3 supports the likelihood that these changes were being incorporated into routine practice rather than representing a transient response to audit alone. This project has been designed and reported in line with recognized standards for QI reporting [14], to support transparency and reproducibility.

## Conclusions

This QI initiative showed that implementation of a standardized febrile neutropenia protocol, multidisciplinary education, rapid-alert communication, early order initiation, vascular access escalation planning, and improved antibiotic readiness was associated with faster and more reliable delivery of empiric antibiotics across successive PDSA cycles. The proportion of patients receiving antibiotics within 60 minutes improved from one-half at baseline to more than four-fifths after intervention, while documentation quality also improved substantially. These findings support the use of structured PDSA-based QI methods to operationalize febrile neutropenia guidelines in routine oncology care and provide a practical model that may be adapted by other centres seeking to improve performance against the 60-minute target.

## Appendices

The bundle comprised seven components, each summarized in Table 2 with its mode of delivery, timing of introduction and responsible personnel. Cycle 1 served as the baseline (pre-intervention) audit. The components were introduced together as a single package at the start of Cycle 2 in the order listed in Table 2. Staff training and protocol reinforcement were repeated after Cycle 2, before Cycle 3. Implementation was coordinated by the project investigators, with unit nurse champions supporting day-to-day adherence in the ED and inpatient oncology ward. Adherence to each component was assessed through a daily structured audit checklist. 

**Table 2:** Components of the Febrile Neutropenia Intervention Bundle FN: febrile neutropenia, IV: intravenous, IDSA: Infectious Diseases Society of America, ASCO: American Society of Clinical Oncology, ED: emergency department

Component and delivery	Responsible staff
1. Standardized FN management protocol and order set (blood cultures plus first-dose empiric IV antibiotic), based on IDSA/ASCO guidance	Investigators
2. Staff training: focused teaching sessions for ED and inpatient staff on the time-sensitivity of FN, the mortality risk of delay, and the bundled cultures-plus-antibiotics approach	Investigators Unit Nurse
3. Fever-alert chart flags to identify febrile oncology patients at the point of care	Unit Nurse
4. Triage prioritization: FN recognized as a distinct chief complaint with a revised ED triage level; patients and caregivers educated to report recent chemotherapy on arrival	ED triage staff
5. Streamlined notification: immediate notification of the treating team on arrival of a febrile oncology patient	ED nurse/Unit Nurse
6. Vascular access escalation algorithm: prompt peripheral IV access when the indwelling catheter cannot be rapidly accessed	Nursing staff
7. Ward-level antibiotic availability: assured stock of first-dose empiric antibiotics in the oncology ward	Pharmacy in-charge
